# The Clinical Handshake: Etiological Co-formulation in the Patient Encounter

**DOI:** 10.1007/s40596-025-02118-6

**Published:** 2025-02-18

**Authors:** Carissa M. Zeleski

**Affiliations:** Boise VA Medical Center, Boise, ID USA

I first encountered George Engel’s famous paper on the biopsychosocial model, “The Need for a New Medical Model: a Challenge for Biomedicine,” while investigating the history of etiological thought in psychiatry. Through a combination of my own interests and strong mentorship, I had read a substantial amount on the mechanisms of psychic pain. I was wondering why, as a field, we had difficulty incorporating the rich array of etiological models into a common language for our clinical work. My mentors acknowledged the issue and presented the simple rationale that cohesion was not possible with so many different camps in opposition to one another. Nonetheless, they encouraged me to develop my thoughts. That brought me to reading Engel because, for all I could tell, the biopsychosocial model was one we could almost all agree on [1].

Reading the paper, I expected a run-down on the way psychopathology is made unique by an individual’s psychology and environment. I was surprised to find that the paper contained something very different: an approach to practicing medicine from the perspective of the physician’s cultural role. To my reading, the fundamental assertion in the paper was that all patients carry “folk” ([2], p.130) beliefs about how health and illness function and that the physician has the cultural task of resolving problems of illness in part by offering “explanations about disease” ([2], p. 130). In this spirit, Engel challenges physicians “to account for the dysphoria and dysfunction which lead individuals to seek medical help” ([2] p. 133) and makes the point that for the psychiatrist this includes discerning a biomedical illness from a problem of living. He points out that in contemporary culture, most folk models are biomedically influenced. He reasons it is “the doctor’s, not the patient’s, responsibility to establish the nature of the problem and whether or not it best be handled in a medical framework” ([2], p. 133).

I held onto this concept while I continued to seek out an entry point for bringing etiological language into my practice. Then, in the summer of 2022, Joanna Moncrieff et al. [3] published “The Serotonin Theory of Depression: a Systematic Umbrella Review of the Evidence.” This paper highlighted the inconsistent evidence around the chemical imbalance theory of major depression. The paper made headlines in the popular press [4]. The concern noted in the discussion section is that patients are formulating their illnesses incorrectly with the “chemical imbalance” ([3], p. 3253) model and using it to make poor decisions which ultimately impair their recovery. The attention that the paper received by the public reflected that very point, as many people were upset to have their folk notions on how to recover from depression upended by a press release. I noted then that both Engel and Moncrieff were addressing the same question: should we care about what the patient believes? For both, the answer is yes.

## The Importance of Etiology

It took repeated readings of Engel’s paper to appreciate the importance he was placing on the cultural role of the physician. I needed to understand the history of the medical model and the philosophy of disease to become attuned to the way that the physician had been culturally relevant far before biomedicine had its heyday. Over time, my perspective shifted to seeing the physician as a cultural figure of great importance regardless of biomedical savvy. In that vein, I became increasingly interested in Engel’s challenge to offer explanations to patients that were meaningful without discarding the benefits of biomedicine [2].

The question then became, how to choose which explanations to offer. The Diagnostic and Statistical Manual (DSM) [5] is not etiological, so while I continued to use it to organize, bill, and prescribe medications, I let go of it as a way to explain. I was well-versed in models, and as I tried applying them it became clear that choosing just one type for all cases was insufficient. There was no doubt that different problems correlated with different models, and that different models served different purposes. Nassir Ghaemi has a similar observation in a 2006 opinion piece, concluding that “no single paradigm can explain all of psychiatry; no dogma is sufficient. Combining all methods on whatever grounds (pragmatic or otherwise) is, however, barely a step forward; more is not better” ([6] p. 622).

Ghaemi is a pluralist, believing we need “a certain method in some circumstance but not others” ([6] p. 622) implying that we should have some parameters for when to use what. This left me asking: what might that circumstance be? With no top-down guidance and inspired by the emphasis on partnership that I had learned from Miller and Rollnick’s motivational interviewing [7] and Sandra Steingard’s collaborative approach for pharmaceutical prescribing in *Critical Psychiatry* [8], I started proposing different models to my patients and getting their feedback. The result was that the patient and I began to work together on a project of collaboration that I now call co-formulation.

## Review of Etiological Models

I think of co-formulation as a kind of clinical handshake. In addition to being a gesture of greeting and agreement, the handshake has two sides: the patient brings their ideas about what is going on to the encounter, and then the physician responds in turn. When I first began co-formulating with patients, I would pull from my personal bank of models that which I thought would be most helpful for them based on how they were thinking about their illness, abandoning my own default way of approaching things. Important first steps are taken here: I release my hold on one reductionist measure for sickness and health and I start to observe the circumstances in which some explanations work better than others. The patient is not harmed, as their alliance grows with this engagement and with that their treatment response [9]. I value both what is most salient to them and what I think is most likely causing the distress.

Under the pressure of sharing my approach with residents, I realized I needed to develop a way to teach etiology that was more constrained than my personal bank. To this end, I developed a framework of ten mechanisms of emotional suffering I use most in clinical practice. Each leads to a reasonable set of actions that becomes the treatment plan. If time is spent in the clinical encounter to build insight, these mechanisms become models for recovery. I have included these in Table 1, alongside the associated presentations where I find they are most useful. It is important to note that the use of the term model here does not imply that no other model can ever be used to understand a situation. Many therapies use mental models that are meaningful and effective, but I would suggest that those models would fall under one of the ten mechanisms and represent zooming in rather than something distinct.

**Table 1:** A ten-point framework of explanatory models

Model	Mechanism	Case elements	Treatment
Evolutionary	The function and dysfunction of the human behavioral system can be determined relative to its effectiveness in the environment of evolutionary adaptedness [10]		
	A functional behavioral system adapted to hunter-gather societies may be maladaptive in the modern day [11]	Over-stimulus Addiction Isolation Overwork	Identify conditions which overtax the nervous system Counsel on lifestyle change
	A dysfunctional behavioral system may arise from genetic trade-offs, tissue breakdown, and senescence [11]	Developmental onset Severe mental illness Brain disease Brain injury Neurocognitive decline	Psychoeducation on neurological differences Obtain appropriate accommodations
Biochemical	Neurotransmitters in excess or deficit cause symptoms	Psychosis Anhedonia Inattention Nightmares Reward-seeking	Describe how medications modify activity in established pathways Counsel on substance use
Diathesis-stress	Physiological triggers can bring latent genetic conditions forward	Schizophrenia Bipolar disorders Seasonal or hormonal pattern Adverse childhood experiences	Accept genetic risks and the need for biological treatment Monitor periods of vulnerability Counsel on substance use
Affective	Primal affects are powerful urges to action [12] which can drive impulsivity or over-suppression	Dysregulation Impulsivity Alexithymia Somatization Distortions Trauma	Bring awareness to the embodied experience of affect Increase tolerance with mindfulness, medication, and therapy
Resilience	A biopsychological factor that increases tolerance to stress [13]	Chronic stress Chronic anxiety Depression Burnout	Give serotonergic medications [14] Provide psychoeducation on resilience
Psychodynamic	Drives and desires outside of conscious awareness can precipitate and perpetuate illness	Internal conflict Childhood trauma Dissociation Conversion Treatment impasse [15]	Bring awareness to the unconscious elements Psychodynamic therapy
Attachment	Secure attachment is important for well-being [16]	Relational conflict Loneliness	Acknowledge needs and normalize difficulties Interpersonal or couples therapy
Compassion and forgiveness	Chronic shame or anger is a barrier to well-being	Poor self-image Resentments Illness stigma	Encourage a practice of mindfulness and self-kindness [17] Emphasize common humanity [17] Encourage openness to spiritual wisdom
Phase of life	Change produces normal grief and anxiety	Loss Life transition Aging Health problems	Normalize suffering and provide emotional support Problem solve
Hierarchy of needs	To reach high-level goals, more basic needs must be met	Intense anxiety Health crisis Unstable housing Food insecurity Lack of safety Marginalization	Acknowledge dysphoria as a normal response Prioritize getting needs met

The framework is an organizational tool intended to map a biopsychosocial territory including medical problems, problems of living, and conditions in between. In effect, the tool allows me to answer Engel’s challenge to “establish the nature of the problem” ([2], p. 133) and “evaluate whatever problems the patient presents and recommend a course of action” ([2], p. 133). However, the framework is an abstraction and is not meant to replace traveling the territory. Evolutionary psychology, affective neuroscience, psychodynamics in medication management, resilience factors, and principles of compassion are areas which may be underexplored in the typical training program. To travel this less familiar territory, I recommend Martin Brüne’s *Textbook of Evolutionary Psychiatry and Psychosomatic Medicine* [18], Jaak Panksepp’s *The Archeology of Mind* [12], David Mintz’s *Psychodynamic Psychopharmacology* [15], and Steven Southwick and Dennis Charney’s *Resilience* [19]. Helpful peer-reviewed articles include “Stress Response Modulation Underlying the Psychobiology of Resilience” by Lynette Averill et al. [13] and “Self-Compassion: An Alternative Conceptualization of a Healthy Attitude Towards Oneself” by Kristin Neff [17].

## Using Etiological Co-formulation

The clinical handshake requires that we invite the patient to extend their hand: we must ask the patient to articulate their thoughts on what makes them feel unwell. In addition to the standard history-taking, asking pointed questions and listening well will elicit the full spectrum of patient folk beliefs about illness. What do they think the problem is? Is their problem a result of their biology? Is it a result of learned responses? Is it due to their environment? Have they been told a diagnosis? Do they believe it? Do they have their own ideas? What does it mean to have that diagnosis? Can they recover? What do they think the medication will do? What were they hoping to get from an encounter with the psychiatrist? What does health look like? Articulating these answers is effortful and leaves the patient vulnerable, so providing encouragement and psychological safety is important. A collection of common folk beliefs that arise in the encounter and how they could be addressed using the models is presented in Table 2.

**Table 2:** Examples of folk beliefs and psychiatric explanations

Model	Folk belief	Psychiatric explanation
Evolutionary	When I feel dysphoric, it means I am malfunctioning	There are other reasons that you could feel dysphoria
	Even though I have serious mental illness I can resolve it on my own	Genetic dysregulation of neurotransmitters means you need medication
	I should be able to handle anything	Our nervous systems were adapted to austere, high-trust environments; we may respond with distress to the modern day
Biochemical	If I have psychosis, it means I am crazy	Dopamine excess could cause psychosis in anyone
	The antipsychotic is not for me because I am not psychotic	Medications are not always specific to one neurotransmitter
Diathesis-stress	I did not always need medications so it can’t be biological	Some genetic illnesses are triggered by physiology or the environment
	Other people can handle stress/substance use so that cannot be the problem	Humans are widely variable. It is important to accept your own individual differences
Affective	My emotions are a source of truth	Affects are primal survival urges that often falsely alarm
	My emotions can be ignored	The body experiences affects, and if you are unconscious to this it comes out in other ways
Resilience	The serotonin reuptake inhibitor (SRI) is supposed to make me happy	SRIs increase neuroplasticity [14] and blunt arousal, but they do not elicit happiness
	The goal of treatment is to be totally happy and relaxed	Treatment often targets learning, endurance and symptom control instead of bliss
	Stress is not that harmful, it is just mental	Stress induces cortisol release which can promote depression [14]
	I cannot seem to have good feelings	Positive feelings can be cultivated through purpose, values-based living, facing fear, optimism, helping others, self-care, social support [19] shared experience, mindfulness and self-kindness [17]
Psychodynamic	Medication only functions biologically	Treatment with medications can bring up old relationship patterns and feelings about taking the sick role [15]
	I cannot tolerate the medications	Rejecting medications can be a way of expressing unconscious anger and autonomy [15]
	The medications do not work	Rejecting recovery can be a way of expressing unconscious shame and guilt [15]
Attachment	I am weak in needing others so badly	You are wise; good relationships are a primary predictor of well-being [16]
	It is good to keep to myself so that I do not upset others	Securely attached partners handle strife together
Compassion and forgiveness	Only crazy people talk to themselves	Talking in a kind way to oneself is healthy
	I am broken because of my illness	Your genetics have strengths as well and you are not alone
Phase of life	Being medically ill has made me mentally ill	You have a normal fear of infirmity and death
	Now that I am older, I am no longer myself	Age comes with opportunities for growth due to changing tasks and roles
Hierarchy of needs	Getting rid of my dysphoria gets rid of the problem	Addressing problems of living and getting needs met is an important part of recovery
	I need to work on myself to achieve my higher purpose	You may need to improve your environment first

In the end, the process is a matter of reaching a handshake agreement about what is going on. The patient needs to endorse the physician’s insights as being salient and ringing true. The physician needs to be reasonably transparent about their interpretations, using the patient’s own words, actions, and values as support. One might think I would prescribe less medications with this process, but I find the handshake allows me to prescribe more. I work in a high acuity setting so I often see multiple mechanisms at play, and there is need for rapid acting support. I apply the DSM’s recommended approach to case formulation, using the criteria and patient history to diagnose, which in turn helps me use the evidence base [5]. In the handshake, I discuss the etiological rationale behind the choice. The merging of the two systems may not be optimal but works better than one would think. Additionally, I am confident that with the handshake the patient will be able to engage in treatment without medications weakening their agency. This is the essence of etiological co-formulation: the expertise and prescribing power of the physician sets the patient on the path to solving their own problem.

I imagine that the collaborative process I describe is familiar to experienced psychiatrists who use shared decision-making models. However, I think extra attention to etiological co-formulation can benefit even these experts. This is because even in shared decision-making a co-formulation aspect is not explicitly required. A 2020 review of shared decision-making articles specific to adults with severe mental illness found that communication was broadly emphasized in the process [20]. However, there is no framework or content provided to support etiological co-formulation. Because shared decision-making models involve many steps and are not specific to psychiatry, it is not surprising that hammering out the elusive details of this single element is a stone unturned.

## Teaching Etiological Models

How do we learn this clinical handshake? I recommend a stepwise approach. First, the learner adopts a mental framework, like the one I propose here, to promote attention to etiology. Second, the learner flags etiological explanations as they encounter them in training and their own independent study, filing them away for clinical use. Finally, the learner practices the co-formulation procedure, applying explanations to the patient presentation by asking the right questions, listening well, presenting information clearly, and collaborating. Currently, in our training program in the first year, I use models at the bedside and run a journal club featuring Engel’s paper. In the second year, residents get education on the etiological framework in didactics. In the third year, they are given a chance to present their cases and have their fourth-year peers formulate with them using various models. This moves the residents through the three steps over time. Additionally in outpatient supervision, I ask my supervisees to be aware of their patient’s internalized folk models and use their time with the patient to build insight. Finally, I run an optional book club where residents can practice verbalizing perspectives on suffering.

Etiological co-formulation is the psychiatrist’s purview. The ability to talk with our patients about what constitutes illness and wellness takes practice far beyond what is covered in non-physician training paths. Four years of supervised psychiatry training provides the opportunity to study the models and practice co-formulation under supervision. In my experience, co-formulation is a powerful way to address the challenges we face in psychiatry today, such as treatment resistance, social media contagions, increasing complexity of diagnosis, and compounding social stress. If psychiatrists can ingrain co-formulation in our professional identity, we can practice the transformative care that the public expects for the costs incurred by our services.

## Data Availability

No new data were generated or analyzed during this study.
